# Causal relationship between lipid-lowering drugs and ovarian cancer, cervical cancer: a drug target mendelian randomization study

**DOI:** 10.1186/s12885-024-12434-z

**Published:** 2024-05-31

**Authors:** Jinshuai Li, Zixian Yang, Tao Wang, Mengqi Li, Xiangjian Wu, Xiaoyan Fu, Chunfeng Yang, Yangpu Li, Ximing Wang, Zhiming Lan, Minfang Li, Sheng Chen

**Affiliations:** 1 The fourth Clinical Medical College of Guangzhou University of Chinese Medicine, Shenzhen, Guangdong 518033 China; 2 Shenzhen Traditional Chinese Medicine Hospital, Shenzhen, Guangdong 518033 China; 3 Jinan University School of Traditional Chinese Medicine, Guangzhou, Guangdong 510632 China; 4 Department of Nutrition and Food Hygiene, School of Public Health, Wuhan University, Wuhan, 430071 China

**Keywords:** HMGCR, PCSK9, Ovarian cancer, Cervical cancer, MR

## Abstract

**Background:**

The causal impact of lipid-lowering drugs on ovarian cancer (OC) and cervical cancer (CC) has received considerable attention, but its causal relationship is still a subject of debate. Hence, the objective of this study is to evaluate the impact of lipid-lowering medications on the occurrence risk of OC and CC through Mendelian randomization (MR) analysis of drug targets.

**Methods:**

This investigation concentrated on the primary targets of lipid-lowering medications, specifically, 3-hydroxy-3-methylglutaryl-coenzyme A reductase (HMGCR) and proprotein convertase kexin 9 (PCSK9). Genetic variations associated with HMGCR and PCSK9 were derived from published genome-wide association study (GWAS) findings to serve as substitutes for HMGCR and PCSK9 inhibitors. Employing a MR approach, an analysis was conducted to scrutinize the impact of inhibitors targeting HMGCR and PCSK9 on the occurrence of OC and CC. Coronary heart disease (CHD) risk was utilized as a positive control, and the primary outcomes encompassed OC and CC.

**Results:**

The findings of the study suggest a notable elevation in the risk of OC among patients treated with HMGCR inhibitors (OR [95%CI] = 1.815 [1.316, 2.315], *p* = 0.019). In contrast, no significant correlation was observed between PCSK9 inhibitors and the occurrence of OC. Additionally, the analysis did not reveal any noteworthy connection between HMGCR inhibitors, PCSK9 inhibitors, and CC.

**Conclusion:**

HMGCR inhibitors significantly elevate the risk of OC in patients, but their mechanism needs further investigation, and no influence of PCSK9 inhibitors on OC has been observed. There is no significant relationship between HMGCR inhibitors, PCSK9 inhibitors, and CC.

**Supplementary Information:**

The online version contains supplementary material available at 10.1186/s12885-024-12434-z.

## Introduction

Ovarian cancer (OC) and cervical cancer (CC) are prevalent gynecological malignancies, contributing significantly to cancer-related fatalities among women globally and profoundly affecting patients’ quality of life [1]. The incidence rates of OC and CC exhibit variations among different countries, but the overall global incidence is escalating, imposing a considerable burden on the world [2]. OC and CC consistently rank among the foremost cancers in women on a global scale, with disparities observed between developed and developing countries [3, 4]. Despite the existence of diverse treatment options, OC and CC often manifest at advanced stages with bleak prognoses. With the continuous progress in medicine, targeted therapy is emerging as a promising avenue [5]. Therefore, it is imperative to investigate potential etiological mechanisms and identify new treatment targets for the effective prevention and treatment of OC and CC. Observational studies hint at an association between OC and CC with lipid abnormalities [6–8], but the risk of OC and CC associated with lipid-lowering treatment remains contentious [9, 10]. Therefore, it is imperative to conduct a thorough assessment of the influence of lipid-lowering medications on OC and CC.

Lipid-lowering medications extensively utilized in clinical settings include inhibitors targeting 3-hydroxy-3-methylglutaryl-coenzyme A reductase (HMGCR). The effectiveness of HMGCR inhibitors in preventing cardiovascular diseases, such as coronary heart disease (CHD), is well-established. Nonetheless, a substantial controversy remains regarding the potential link between the use of HMGCR inhibitors and the occurrence of OC and CC as indicated in observational studies [9–14]. In vitro studies suggest that statins, a subtype of HMGCR inhibitors, can inhibit the proliferation of OC [15], induce apoptosis in CC cells [16], moreover, they demonstrate anti-metastatic and anti-tumor characteristics by activating mitogen-activated protein kinase, as highlighted in previous research [17]. Observational studies are prone to inherent confounding factors, potentially leading to inconsistent outcomes. Essentially, we cannot dismiss the suspicion that statins might have a latent impact on OC and CC.

In contrast, the efficacy of lowering cholesterol levels is achieved by proprotein convertase kexin 9 (PCSK9) inhibitors, which enhance the expression of low-density lipoprotein receptor (LDLR). The latest research developments highlight variances in PCSK9 expression levels observed between normal and tumor cells [18]. PCSK9 is involved in the regulation of diverse proteins and signaling pathways in cancer [19]. This is substantiated by a Mendelian randomization (MR) study that affirms a significant decrease in the risk of prostate cancer with PCSK9 inhibition [20]. Experiments conducted with in vitro models of human lung adenocarcinoma cells illustrate that inhibiting PCSK9 induces apoptosis, demonstrating noteworthy anti-tumor activity [21]. Recent findings suggest that inhibiting PCSK9 enhances the tumor’s response to immune checkpoint therapy, and both genetic and pharmacological reduction of PCSK9 can suppress tumor growth [22]. The cholesterol-lowering effect of PCSK9 inhibitors is considered a potential mechanism against cancer [23]. However, there is limited reporting on the relationship between PCSK9 inhibitors and OC and CC, necessitating exploration of their association.

The analysis of drug targets using MR involves the use of genetically simulated variations to emulate the pharmacological inhibition of drug targets, acting as instrumental variables(IVs). Through regression analysis, this method elucidates causal inferences regarding the influence of drug-gene targets on specific outcomes, aiding in the estimation of enduring effects arising from drug utilization [24]. The utilization of genetic variation aims to minimize the influence of confounding factors on estimates. In this investigation, we employed MR analysis of drug targets to investigate the causal link between genetically predicted suppression of HMGCR and PCSK9 and their correlation with OC and CC.

## Methods

### Selection of HMGCR and PCSK9 IVs

**Fig. 1:**
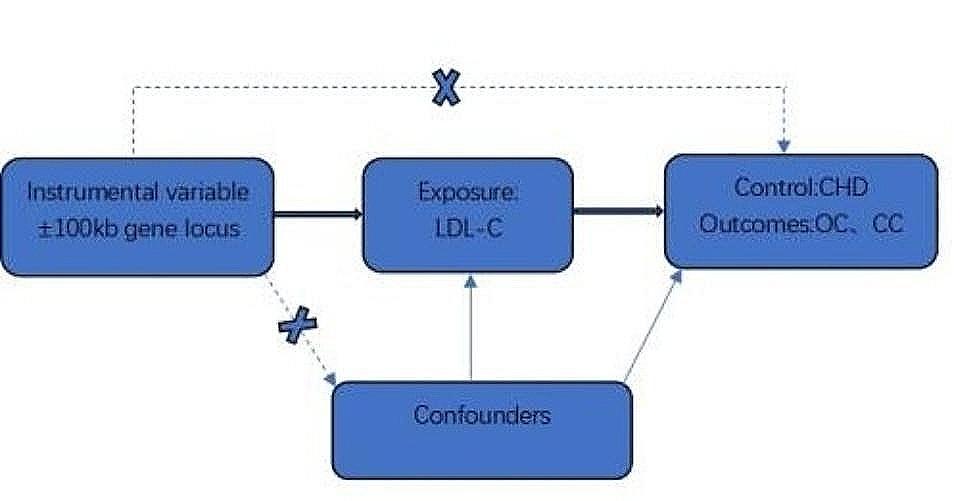
Study overview and design of MR analysis of drug targets

In this study, the genome-wide association study (GWAS) data for low-density lipoprotein cholesterol (LDL-C) were obtained from the Medical Research Council - Integrative Epidemiology Unit (MRC-IEU) GWAS database (https://gwas.mrcieu.ac.uk/) under the GWAS ID ieu-b-4846, covering 70,814 European individuals. IVs aimed at lowering LDL-C through the targeting of HMGCR and PCSK9 were obtained to mimic the impacts of HMGCR inhibitors and PCSK9 inhibitors [25]. The choice of IVs was determined by their notable genome-wide association with LDL-C (*P* < 5e-08) and their positioning within ± 100 kb of the HMGCR or PCSK9 loci (refer to Fig. 1). To alleviate the influence of substantial linkage disequilibrium (LD) on the findings, a threshold for LD (r2 < 0.3) was implemented [26]. An F-statistic greater than 10 indicated a robust correlation between single nucleotide polymorphism (SNP) and exposure; hence, SNPs with an F-statistic greater than 10 were preserved, with the calculation of the F-statistic done using the formula F = Beta2/SE2 [27]. Assumptions in MR necessitated that SNPs were not directly linked with the result and did not impact the outcome through confounding factors beyond the exposure. Consequently, PhenoScanner (http://www.phenoscanner.medschl.cam.ac.uk/) [28] was used to exclude SNPs directly linked to the outcome and simultaneously eliminate SNPs correlated with confounding factors for the outcome. Presently identified risk factors for OC encompass older age, genetics, family history, hormone replacement therapy, nulliparous motherhood, and dietary fat [29]. In contrast, acknowledged risk factors for CC involve the use of oral contraceptives, infection with Chlamydia trachomatis, Intra-uterine device use, endometriosis, vitamin A, carotene and vitamin E [30]. . Six significant SNPs for HMGCR and 12 significant SNPs for PCSK9 were retained (Supplementary Table S1). Employing another dataset from the Global Lipids Genetics Consortium (GLGC) [31], the aforementioned procedure was reiterated to obtain IVs for HMGCR and PCSK9, ensuring result stability. This dataset primarily comprised European populations, preserving 5 significant SNPs for HMGCR and 8 significant SNPs for PCSK9 (Supplementary Table S2).We employed existing research data, and the initial study received approval from the relevant ethics committee.

### Source of result data

We utilized OC and CC as outcomes for the MR analysis, with CHD serving as a positive control, encompassing 22,233 cases and 64,762 controls. All datasets were sourced from European populations and are available in the MRC-IEU GWAS database (https://gwas.mrcieu.ac.uk/), where the GWAS IDs are as follows: coronary heart disease GWAS ID - ieu-a-8; OC GWAS ID - ebi-a-GCST90018888; CC GWAS ID - ebi-a-GCST90018817.

### Data analysis

HMGCR and PCSK9 inhibitors are crucial in the clinical treatment of CHD. To confirm the efficacy of IVs, we utilized CHD GWAS data as a positive control. Initially, we matched the drug-targeting IVs related to exposure with the outcome dataset, excluding SNPs exhibiting palindromic structures [32]. Subsequently, analyses were performed using inverse variance weighting (IVW), weighted median, MR Egger, simple mode, and weighted mode.The IVW method is the most commonly used [33] and considered crucial in MR analysis. Thus, when discrepancies arise in the findings from these approaches, the IVW method is given precedence as the primary discovery method. Heterogeneity tests were conducted using MR Egger and IVW methods. Cochran’s Q-value was employed to assess heterogeneity in genetic instruments, with *p* > 0.05 indicating no significant heterogeneity. In the presence of heterogeneity, the IVW random-effects model was used to mitigate its impact. If no heterogeneity was observed, the inverse variance-weighted fixed-effects model (IVW_FE_) was utilized. MR Egger regression equations and MR-PRESSO were employed to evaluate horizontal pleiotropy of genetic instruments, with *p* > 0.05 indicating no horizontal pleiotropy [34]. The MR-PRESSO test can examine the presence of outliers, and if outliers are identified, MR analysis is repeated after their removal. The analysis of data was carried out utilizing the MRPRESSO and TwoSampleMR packages in R version 4.3.2 [34, 35].

## Results

### Positive control analysis

**Fig. 2:**
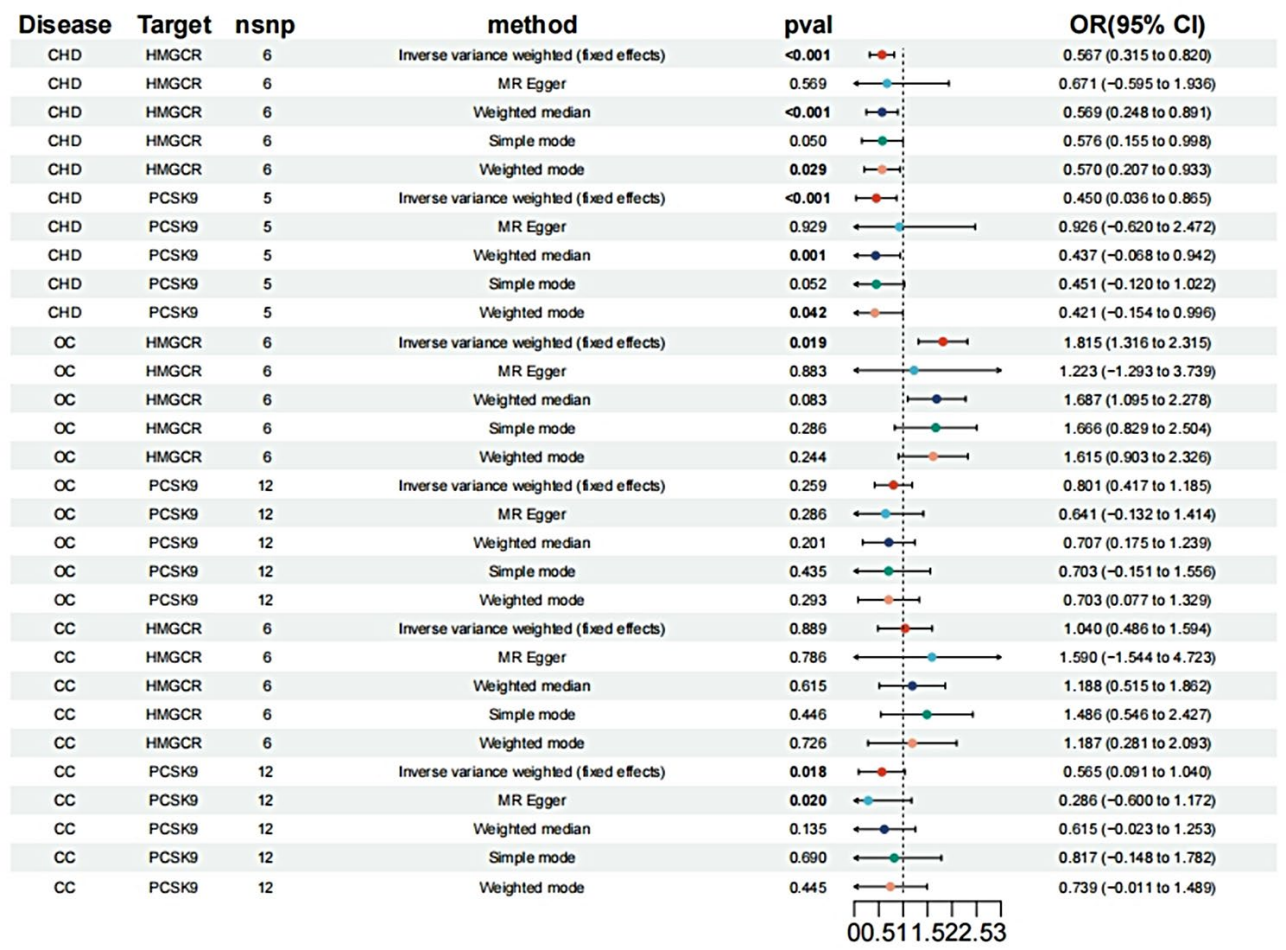
Effects of HMGCR and PCSK9 inhibitors on CHD, OC, and CC

**Fig. 3:**
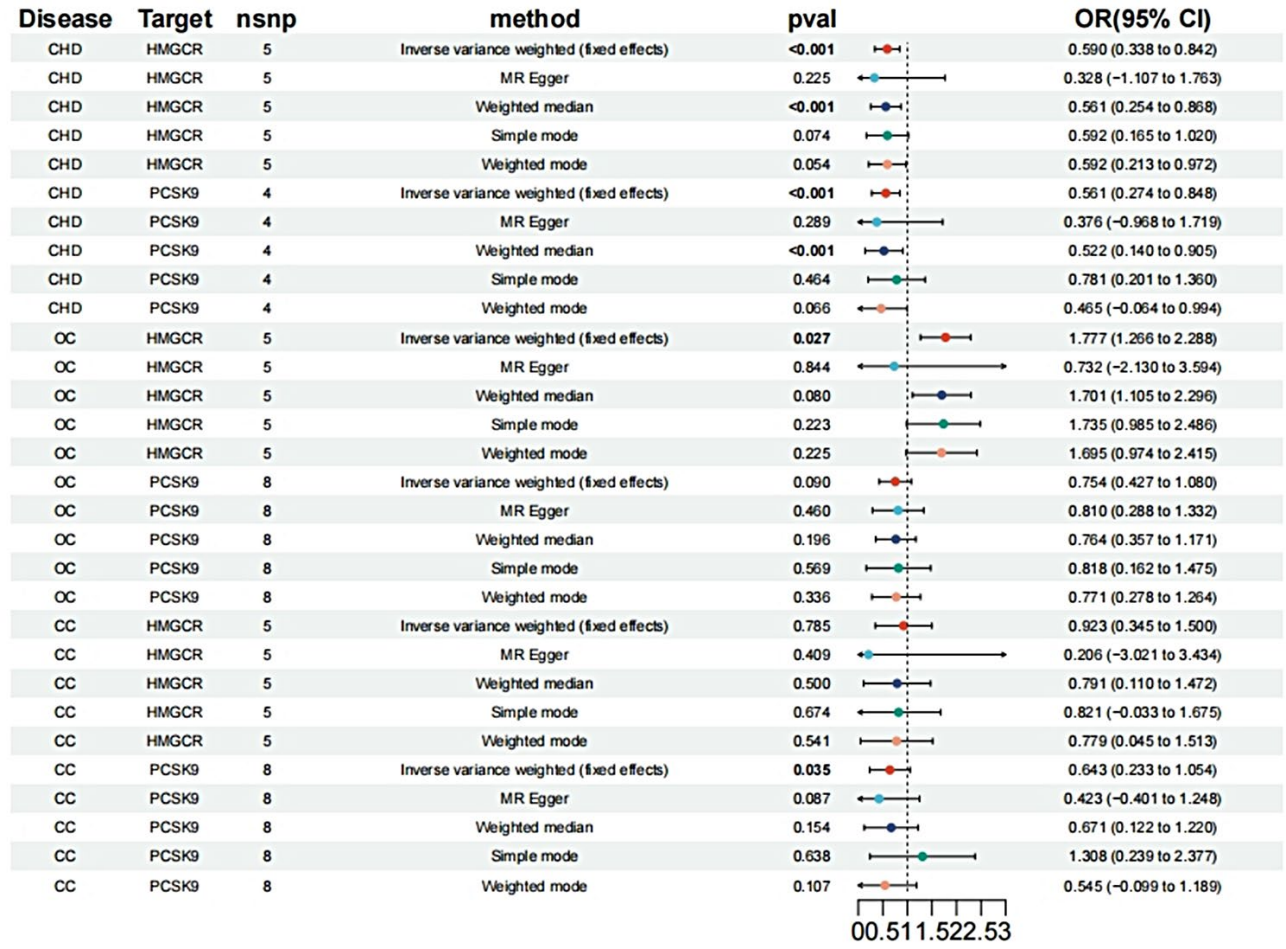
Repeated analyses were conducted to assess the effects of HMGCR and PCSK9 inhibitors on CHD, OC, and CC

The IVW analysis results reveal that HMGCR inhibitors (OR [95%CI] = 0.567 [0.315, 0.820], *p* < 0.001) and PCSK9 inhibitors (OR [95%CI] = 0.450 [0.036, 0.865], *p* < 0.001) both significantly reduce the risk of CHD. Furthermore, the results of the other four methods are shown below.

(Fig. 2). Similar findings were replicated in a subsequent analysis using an additional GWAS dataset from GLGC, where HMGCR inhibitors (OR [95%CI] = 0.590 [0.338, 0.842], *p* < 0.001) and PCSK9 inhibitors (OR [95%CI] = 0.561 [0.274, 0.848], *p* < 0.001) showed consistent effects (Fig. 3).

### Causal relationships of HMGCR and PCSK9 inhibitors with OC and CC

According to the findings from the IVW analysis, HMGCR inhibitors have been recognized as a potential factor associated with the risk of OC(OR [95%CI] = 1.815 [1.316, 2.315], *p* = 0.019) (Fig. 2), while no significant relationship is observed between PCSK9 inhibitors and OC (Fig. 2). Furthermore, there is no association found between HMGCR inhibitors, PCSK9 inhibitors, and CC (Fig. 2). To validate these findings, we repeated the analysis using an alternative GWAS dataset from GLGC, which also suggests that HMGCR inhibitors increase the risk of OC (OR [95%CI] = 1.777 [1.266, 2.288], *p* = 0.027) (Fig. 3). In contrast, PCSK9 inhibitors do not exhibit a significant association with OC in this analysis (Fig. 3). Similarly, no association is observed between HMGCR inhibitors, PCSK9 inhibitors, and CC (Fig. 3).

### Sensitivity analysis

We assessed heterogeneity using Cochrane’s Q test, and no significant heterogeneity was observed (Supplementary Table S3). Consequently, we employed the IVWFE method in our MR analysis. To evaluate horizontal pleiotropy, we utilized MR-Egger regression and MR-PRESSO global test methods, both of which did not indicate the presence of horizontal pleiotropy (Supplementary Table 3). The robustness of the results was further supported by leave-one-out analysis (Supplementary Fig. S4、S5、S6). To ensure result consistency, we repeated the procedures with GWAS data from GLGC, and no heterogeneity was observed (Supplementary Table S4). Therefore, we continued using the IVWFE method in our MR analysis, which also did not detect pleiotropy (Supplementary Table S4).

## Discussion

In this extensive MR analysis, encompassing 1,588 cases of OC and 244,932 controls, as well as 909 cases of CC and 238,249 controls, We explored the influence of two prevalent LDL-C-lowering drug targets (HMGCR and PCSK9) on the risk of OC and CC. Our findings indicate that, genetically, HMGCR inhibitors pose a risk for OC, whereas PCSK9 inhibitors are not significantly associated with OC. Furthermore, neither HMGCR inhibitors nor PCSK9 inhibitors exhibit a significant association with CC.

In recent years, observational studies have explored the impact of lipid-lowering drugs on OC and CC risk, yielding inconsistent results [9–14]. A meta-analysis, incorporating literature up to July 2021, suggests a potential preventive effect of statin drugs on OC, indicating a reduction in risk [9]. Another meta-analysis involving 14 observational studies also supports a negative correlation between statin use and OC risk, although no significant association is found with CC [13]. These findings hint at the potential protective role of statins against OC. However, a cohort study contradicts this, failing to establish a reduction in OC risk with statin use but indicating a decreased risk of CC [12]. Another extensive retrospective study similarly fails to find a clear association between statin use and OC [14]. Some research even proposes that statins might increase OC risk [10], aligning with our study results. PCSK9 exhibits distinct expression in normal and tumor cells [18], and in in vitro studies of lung adenocarcinoma, inhibiting PCSK9 demonstrates anti-tumor activity [21]. In vitro research implies that inhibiting PCSK9 may impact the survival of OC cells [36], but clinical observational studies on PCSK9 inhibitors and OC, CC are yet to be published. Lipid-lowering drugs are widely used in clinical settings to prevent various cardiovascular diseases. In recent years, these drugs have shown potential anti-tumor effects. However, considerable debate surrounds the risk of OC and CC with HMGCR inhibitors, and notably, research on the association between PCSK9 inhibitors and OC, CC is lacking. Hence, it is vital to clarify the connection between lipid-lowering medications and OC as well as CC. Observational studies might be influenced by various inevitable confounding elements, leading to varied results. MR studies have become increasingly popular due to their ability to offer a genetic viewpoint on the connection between exposure and outcome, effectively reducing the influence of confounding factors [37].

Our findings reveal a significant increase in OC risk with HMGCR inhibitors, while HMGCR inhibitors show no link to CC risk, suggesting potential long-term side effects. Gene expression analysis [38] and Bonome microarray data [39] demonstrate a significant downregulation of HMGCR in OC. Furthermore, investigations utilizing tissue microarray analysis reveal that OC patients with elevated HMGCR expression demonstrate a markedly improved prognosis [40]. Online survival analysis also highlights a favorable prognosis for patients with higher HMGCR expression [41]. Immunohistochemical results reveal lower HMGCR expression in platinum-resistant OC patients. Collectively, these studies propose that HMGCR contributes to the onset, progression, and prognosis of OC. In conjunction with our study results, PCSK9 inhibitors show no significant influence on OC. We have reason to believe that the HMGCR-OC relationship is not achieved through lowering LDL-C levels, although the specific mechanism remains unknown. A recently published MR study [42], examining three statin drugs as exposure factors and outcomes, including various cancers such as OC and CC, indicates an absence of a noteworthy correlation between the utilization of statin drugs and OC. While this might seem contradictory to our study, the usage of statin drugs does not equate to the inhibition of HMGCR. Additional investigations are required to clarify the precise mechanism through which HMGCR influences OC.

Presently, research exploring the connection between PCSK9 and OC and CC is limited. Despite recent discoveries indicating atypical PCSK9 expression in various cancers [43], such as gastric cancer [44] and breast cancer [45], inhibiting PCSK9 has been documented to elevate cell surface levels of major histocompatibility complex class I and impede tumor growth in cancer cells [46]. Furthermore, a recent MR study indicates a significant reduction in the risk of breast cancer and lung cancer with PCSK9 inhibitor use [47]. Regrettably, our study did not identify a connection between PCSK9 inhibitors and OC, CC.

Our study possesses several strengths. Firstly, it is the inaugural MR study focusing on the relationship between HMGCR inhibitors, PCSK9 inhibitors, and OC, CC. Secondly, our research effectively mitigates the impact of confounding factors, utilizing CHD as a positive control. Sensitivity analysis further substantiates the robustness of our findings. Finally, the identification of HMGCR inhibitors as a risk factor for OC may pave the way for novel approaches in future OC drug development. Although the specific mechanism is currently unclear, for patients with high LDL-C, other lipid-lowering therapies can be considered instead of HMGCR inhibitors, which can minimize the risk of OC while maintaining cardiovascular protection. However, our study also has some limitations. First, our data set uses data from Europeans, which may not be suitable for ethnic groups other than the European population. Second, our study only represents the effect of lifelong inhibition of drug targets on the disease. As for the long-term effects, the relationship between short-term medication and disease risk is still unknown, and our study can only explain the causal effects of exposure and outcome from a genetic perspective, but this study cannot explore the specific mechanism. Finally, our study only represents the causal effects of HMGCR inhibitors, PCSK9 inhibitors and disease risk. The causal effects of long-term medication and disease development and prognosis are also unknown.

## Conclusion

The inhibition of HMGCR significantly increases the risk of OC in patients. However, extensive foundational research and randomized controlled trials are needed in the future to validate these mechanisms. The influence of PCSK9 inhibitors on OC has not been observed. There is no significant association between HMGCR inhibitors, PCSK9 inhibitors, and CC.

### Electronic supplementary material

Below is the link to the electronic supplementary material.

Supplementary Material 1

## Data Availability

Data is provided within the manuscript or supplementary information files.

## Abbreviations

OC: Ovarian cancer
CC: Cervical cancer
CHD: Coronary heart disease
HMGCR: 3-hydroxy-3-methylglutaryl coenzyme A reductase
PCSK9: Proprotein convertase kexin 9
GWAS: Genome-wide association studies
SNP: Single nucleotide polymorphism
SNPs: Single nucleotide polymorphisms
MR: Mendelian randomization
IVW: Inverse variance weighted
IVW_FE_: Fixed effects model with inverse variance weighting
IVs: Instrumental variables
GLGC: Global lipids genetics consortium
LDLR: Low-density lipoprotein receptor
LDL-C: Low-density lipoprotein cholesterol
LD: Linkage disequilibrium
