# Supplementary material for: Causal relationship between lipid-lowering drugs and ovarian cancer, cervical cancer: a drug target mendelian randomization study
Source: BMC Cancer. 2024 May 31;24:667. doi: 10.1186/s12885-024-12434-z (PMC11143665; doi:10.1186/s12885-024-12434-z)
Supplement: Supplementary file 1 — Supplementary Material 1 [file 12885_2024_12434_MOESM1_ESM.docx]

**Supplementary Table1** The detail of instrumental variable corresponding to HMGCR and PCSK9 .

|  | SNP | chr | pos | Beta | SE | *p*-value | Effect alleles | Other alleles |
| --- | --- | --- | --- | --- | --- | --- | --- | --- |
| HMGCR | rs10051965 | 5 | 74560487 | 0.0471 | 0.0053 | 9.61E-19 | T | C |
| HMGCR | rs72633962 | 5 | 74569028 | 0.0634 | 0.0077 | 2.14E-16 | C | T |
| HMGCR | rs2006760 | 5 | 74562029 | 0.0438 | 0.0064 | 7.02E-12 | G | C |
| HMGCR | rs6453131 | 5 | 74644706 | 0.0672 | 0.0053 | 1.82E-37 | G | T |
| HMGCR | rs10041470 | 5 | 74749639 | 0.0644 | 0.0077 | 8.12E-17 | C | G |
| HMGCR | rs3733907 | 5 | 74665860 | 0.0387 | 0.007 | 2.96E-08 | C | G |
| PCSK9 | rs3001844 | 1 | 55494735 | 0.0322 | 0.0051 | 2.21E-10 | C | T |
| PCSK9 | rs2479409 | 1 | 55504650 | -0.0462 | 0.0054 | 9.59E-18 | A | G |
| PCSK9 | rs530804537 | 1 | 55583210 | -0.1945 | 0.0252 | 1.31E-14 | A | G |
| PCSK9 | rs11810371 | 1 | 55496861 | -0.0669 | 0.0121 | 3.64E-08 | A | G |
| PCSK9 | rs2483205 | 1 | 55518316 | -0.0288 | 0.0052 | 2.57E-08 | T | C |
| PCSK9 | rs693668 | 1 | 55521109 | 0.051 | 0.0053 | 7.70E-22 | A | G |
| PCSK9 | rs67171713 | 1 | 55590015 | 0.073 | 0.0127 | 8.32E-09 | C | T |
| PCSK9 | rs11591147 | 1 | 55505647 | -0.3619 | 0.0217 | 3.08E-62 | T | G |
| PCSK9 | rs2479410 | 1 | 55505861 | -0.0408 | 0.0054 | 5.35E-14 | A | G |
| PCSK9 | rs4927191 | 1 | 55491702 | -0.0646 | 0.006 | 9.54E-27 | C | T |
| PCSK9 | rs72909541 | 1 | 55494301 | -0.0602 | 0.011 | 4.49E-08 | T | C |
| PCSK9 | rs10493176 | 1 | 55538552 | -0.0525 | 0.0093 | 1.36E-08 | G | T |

HMGCR, 3-hydroxy-3-methylglutaryl coenzyme A reductase;PCSK9, proprotein convertase subtilisin/kexin 9

**Supplementary Table2** The detail of instrumental variable corresponding to HMGCR and PCSK9 for repeated analysis.

|  | SNP | chr | pos | Beta | SE | p-value | Effect alleles | Other alleles |
| --- | --- | --- | --- | --- | --- | --- | --- | --- |
| HMGCR | rs72633962 | 5 | 74569028 | 0.06 | 0.0072 | 3.53E-15 | C | T |
| HMGCR | rs10515198 | 5 | 74641560 | 0.0583 | 0.0085 | 1.27E-10 | A | G |
| HMGCR | rs6453131 | 5 | 74644706 | 0.0687 | 0.0052 | 3.39E-35 | G | T |
| HMGCR | rs4704219 | 5 | 74748505 | 0.0613 | 0.0052 | 1.42E-28 | T | C |
| HMGCR | rs13356670 | 5 | 74673707 | 0.0391 | 0.006 | 8.12E-10 | G | A |
| PCSK9 | rs644000 | 1 | 55511995 | -0.0597 | 0.0055 | 1.95E-25 | G | A |
| PCSK9 | rs11206510 | 1 | 55496039 | -0.0905 | 0.0066 | 4.96E-39 | C | T |
| PCSK9 | rs2479409 | 1 | 55504650 | -0.0588 | 0.0054 | 6.48E-25 | A | G |
| PCSK9 | rs625619 | 1 | 55518166 | 0.0425 | 0.0053 | 7.64E-14 | A | G |
| PCSK9 | rs7552841 | 1 | 55518752 | 0.0352 | 0.0053 | 1.71E-10 | T | C |
| PCSK9 | rs11591147 | 1 | 55505647 | -0.497 | 0.018 | 1.57E-142 | T | G |
| PCSK9 | rs540796 | 1 | 55524197 | 0.0744 | 0.007 | 4.99E-24 | G | A |
| PCSK9 | rs2483205 | 1 | 55518316 | -0.0514 | 0.0053 | 5.13E-20 | T | C |

HMGCR, 3-hydroxy-3-methylglutaryl coenzyme A reductase;PCSK9, proprotein convertase subtilisin/kexin 9

**Supplementary Table3** The result of heterogeneity test and horizontal pleiotropic test.

| Target | Outcome | Test | Method | P |
| --- | --- | --- | --- | --- |
| HMGCR | CHD | Heterogeneity | Cochran's Q test | 0.823(QMR Egger) |
| HMGCR | CHD | Heterogeneity | Cochran's Q test | 0.902(QIVW) |
| HMGCR | CHD | Pleiotropy | MR-Egger regression | 0.804 |
| HMGCR | CHD | Pleiotropy | MR-PRESSO global test | 0.918 |
| PCSK9 | CHD | Heterogeneity | Cochran's Q test | 0.885(QMR Egger) |
| PCSK9 | CHD | Heterogeneity | Cochran's Q test | 0.818(QIVW) |
| PCSK9 | CHD | Pleiotropy | MR-Egger regression | 0.413 |
| PCSK9 | CHD | Pleiotropy | MR-PRESSO global test | 0.872 |
| HMGCR | OC | Heterogeneity | Cochran's Q test | 0.984(QMR Egger) |
| HMGCR | OC | Heterogeneity | Cochran's Q test | 0.993(QIVW) |
| HMGCR | OC | Pleiotropy | MR-Egger regression | 0.769 |
| HMGCR | OC | Pleiotropy | MR-PRESSO global test | 0.986 |
| PCSK9 | OC | Heterogeneity | Cochran's Q test | 0.192(QMR Egger) |
| PCSK9 | OC | Heterogeneity | Cochran's Q test | 0.219(QIVW) |
| PCSK9 | OC | Pleiotropy | MR-Egger regression | 0.503 |
| PCSK9 | OC | Pleiotropy | MR-PRESSO global test | 0.240 |
| HMGCR | CC | Heterogeneity | Cochran's Q test | 0.294(QMR Egger) |
| HMGCR | CC | Heterogeneity | Cochran's Q test | 0.412(QIVW) |
| HMGCR | CC | Pleiotropy | MR-Egger regression | 0.800 |
| HMGCR | CC | Pleiotropy | MR-PRESSO global test | 0.502 |
| PCSK9 | CC | Heterogeneity | Cochran's Q test | 0.996(QMR Egger) |
| PCSK9 | CC | Heterogeneity | Cochran's Q test | 0.918(QIVW) |
| PCSK9 | CC | Pleiotropy | MR-Egger regression | 0.104 |
| PCSK9 | CC | Pleiotropy | MR-PRESSO global test | 0.920 |

**Supplementary Table4** The result of heterogeneity test and horizontal pleiotropic test for repeated analysis.

| Target | Outcome | Test | Method | P |
| --- | --- | --- | --- | --- |
| HMGCR | CHD | Heterogeneity | Cochran's Q test | 0.671(QMR Egger) |
| HMGCR | CHD | Heterogeneity | Cochran's Q test | 0.697(QIVW) |
| HMGCR | CHD | Pleiotropy | MR-Egger regression | 0.475 |
| HMGCR | CHD | Pleiotropy | MR-PRESSO global test | 0.771 |
| PCSK9 | CHD | Heterogeneity | Cochran's Q test | 0.188(QMR Egger) |
| PCSK9 | CHD | Heterogeneity | Cochran's Q test | 0.265(QIVW) |
| PCSK9 | CHD | Pleiotropy | MR-Egger regression | 0.604 |
| PCSK9 | CHD | Pleiotropy | MR-PRESSO global test | 0.331 |
| HMGCR | OC | Heterogeneity | Cochran's Q test | 0.967(QMR Egger) |
| HMGCR | OC | Heterogeneity | Cochran's Q test | 0.958(QIVW) |
| HMGCR | OC | Pleiotropy | MR-Egger regression | 0.581 |
| HMGCR | OC | Pleiotropy | MR-PRESSO global test | 0.966 |
| PCSK9 | OC | Heterogeneity | Cochran's Q test | 0.672(QMR Egger) |
| PCSK9 | OC | Heterogeneity | Cochran's Q test | 0.762(QIVW) |
| PCSK9 | OC | Pleiotropy | MR-Egger regression | 0.740 |
| PCSK9 | OC | Pleiotropy | MR-PRESSO global test | 0.777 |
| HMGCR | CC | Heterogeneity | Cochran's Q test | 0.972(QMR Egger) |
| HMGCR | CC | Heterogeneity | Cochran's Q test | 0.896(QIVW) |
| HMGCR | CC | Pleiotropy | MR-Egger regression | 0.423 |
| HMGCR | CC | Pleiotropy | MR-PRESSO global test | 0.917 |
| PCSK9 | CC | Heterogeneity | Cochran's Q test | 0.216(QMR Egger) |
| PCSK9 | CC | Heterogeneity | Cochran's Q test | 0.167(QIVW) |
| PCSK9 | CC | Pleiotropy | MR-Egger regression | 0.266 |
| PCSK9 | CC | Pleiotropy | MR-PRESSO global test | 0.202 |
